# Health Literacy Among University Students: A Systematic Review of Cross-Sectional Studies

**DOI:** 10.3389/fpubh.2021.680999

**Published:** 2022-01-21

**Authors:** Lucas Kühn, Philip Bachert, Claudia Hildebrand, Jule Kunkel, Jörg Reitermayer, Hagen Wäsche, Alexander Woll

**Affiliations:** ^1^Institute of Sports and Sports Science, Karlsruhe Institute of Technology, Karlsruhe, Germany; ^2^Central Scientific Institution for Key Competencies, Karlsruhe Institute of Technology, Karlsruhe, Germany

**Keywords:** health literacy, university students, health-promoting universities, systematic review, determinants of health behavior

## Abstract

**Objective:**

The aim of this systematic review was to provide an overview of cross-sectional studies that examined health literacy among university students and to identify possible determinants related to health literacy.

**Method:**

The current review was conducted according to the Preferred Reporting Items for Systematic Reviews and Meta-Analyses (PRISMA). Three databases (PubMed, Scopus, and Web of Science) were systematically searched for cross-sectional studies that examined health literacy among university students. Results of included studies were narratively summarized.

**Results:**

The systematic review includes twenty-one research studies. The majority of studies report health literacy scores among university students that are lower compared to reference samples. The health literacy of students is influenced by different variables (age, gender, number of semesters, course of studies/curriculum, parental education, and socioeconomic background).

**Discussion:**

Health literacy activities should target all students. Universities should make use of their resources and offer health literacy courses for students in which content is used from disciplines available at the university (e.g., medicine, health, or psychology). To increase effectiveness, health literacy courses should be adapted according to the different needs and characteristics of the student subgroups.

## Introduction

University students worldwide experience a high level of psychological stress that exceeds the level of non-students and physiological and psychological health problems (1, 2). The reasons for this are academic responsibilities, financial worries, and adaptation to new life circumstances. These conditions can harm the health of the students (2, 3). To counteract this, the Okanagan Charter for health-promoting universities and colleges (4) was created. Educational institutions that follow the idea of the charter, create campus cultures of wellbeing, equity, social justice, and improve the health of the people who live, learn, and work there. Furthermore, they also strengthen the ecological, social, and economic sustainability of their communities and the society as a whole, considering the responsibility students will later bear in their given environment.

It is important to stress that if people have to achieve their full health potential, they must also take control of its determinants (5). Health promotion is therefore defined by the Ottawa Charter (6) as a process that enables people to better control and promote their health on their own. This idea of empowerment can among other things be accomplished through the improvement of health literacy. The approach of promoting health literacy is indeed deeply rooted in health promotion *per se*: to empower people in a setting to make better decisions about their health and lives in general. A review showed that low health literacy is associated with poorer ability to understand and follow medical advice, poorer health outcomes, and differential use of some healthcare services (7). Educational institutions, such as universities, have the opportunity to optimize the health literacy of their students and empower them to make informed decisions for themselves and their environment (8).

According to Nutbeam (9), health literacy can be divided into three levels: functional, interactive, and critical health literacy. All three levels together comprise complex skills that enable an individual to extract, evaluate, and apply health-related information. Since the WHO introduced the concept of health literacy internationally in the glossary of health promotion (10), more and more definitions have been developed. Parker (11) defines health literacy as a relational concept that, while dependent on individual skills and abilities of a person, is also determined by the demands and complexity of health information and tasks. The most commonly used definitions of health literacy have been compiled by Sørensen et al. (12). In summary, all definitions address the importance of cognitive skills and competent skills that enable obtaining, understanding, and using health information.

There are a variety of reviews on health literacy in diverse populations and professional groups, such as men (13), older adults (14), immigrants (15), and librarians (16). The aim of this systematic review was to provide an overview of cross-sectional studies that examined health literacy among university students and to identify possible determinants. Additionally, we aimed to find out which theoretical frameworks and which different scales were used. Accordingly, the purpose of this review is 2-fold. First, we want to assess the state of research in this field and, second, we intend to identify starting points for decision-makers and health promoters at universities implementing health literacy interventions and adapting them to the needs of the target group.

With the specific target group of students, digital media should be highlighted as an especially relevant source of information, such as health information (17). However, skills required to collect information via the internet differ from those required to collect information from print media, e.g., books (18). Therefore, the definition of eHealth literacy will also be taken into account for this systematic review. It combines health literacy with media and computer-related skills (19).

## Methods

For the purpose of this systematic review, we followed the guidelines described in the Preferred Reporting Items for Systematic Reviews and Meta-Analyses (PRISMA) statement (20). A review protocol has been prepared and can be requested from the authors. The study characteristics used to decide whether a study was eligible for inclusion in the review can be found below: cross-sectional studies (study design) examining the health literacy (outcome) of students in tertiary education of any age (population) and published since the publication of the Okanagan-Charter in 2015 were included in the review. No health status restrictions were imposed. The outcome variables of interest are health literacy and related influencing factors. The health literacy definition of Nutbeam (9, 21) and common health literacy definitions (12) were used as a guiding principle in that respect. Regarding eHealth literacy, the definition of Norman and Skinner (19) served as a decisive criterion. In the studies, the outcome variables had to be given either as primary or secondary outcome variables. Studies were identified by searching three electronic databases (PubMed, Scopus, and Web of Science). The last search was run on February 19, 2020. Additionally, at the end of the search process, the already qualified studies were checked for additional relevant references. Combinations of the following keywords were used to search the databases: university; college; students; adolescents; health literacy; eHealth literacy. The search term was based on the review of Chesser et al. (22), which has a comparable research question but with regard to a different target population. Studies published in English and German were considered for this review. The complete search query can be found in the Appendix (see **“Search term”**). The selection process (title, abstract, and full text) of the studies was conducted by two authors.

A data extraction sheet based on the patient/population, intervention, comparison and outcomes (PICOS) model was used to extract the desired data. Data items were [1] study-relevant information consisting of the name of the study, corresponding authors, the year of publication, and the country, [2] characteristics of participants (e.g., age, gender, study program, and course of studies), the underlying setting (university, college), [3] information on the outcome variables consisting of the theoretical background and the assessment instruments used, and [4] information on the results of the study regarding the health literacy of students and its determinants. The data extraction was always performed independently by at least two authors. Any discrepancies between the authors were resolved through discussion until consensus was reached.

The Appraisal Tool for Cross-Sectional Studies (AXIS) was used to assess the risk of bias of the included studies (23). Two authors independently assessed the quality of the studies. In case of disagreement, another author was consulted, and discussions were held until a consensus was reached. A scoring method was adapted to quantify the risk of bias in individual studies (24, 25). According to this method, studies were categorized as very low risk of bias if they scored at least 19 of 20 questions correctly, as low risk of bias if they scored 17 or 18 out of 20 of the questions of the tool; as the moderate risk of bias if they scored 15 or 16 out of 20, and as high risk of bias if studies scored 14 or fewer points.

The narrative synthesis was based on data synthesis guidelines (26). First, a preliminary synthesis was developed, including initial descriptions of the results of the studies used, grouping the studies according to the PICOS scheme, preparing data and putting them into a common descriptive format, and identifying patterns along with the studies. Subsequently, relationships of the data within and between the studies were investigated. Overall health literacy, various factors that could contribute to health literacy and limitations and practical implications were identified. Also, plausible explanations were developed for the differences found within (characteristics) and between (results) the studies.

## Results

**Figure 1:**
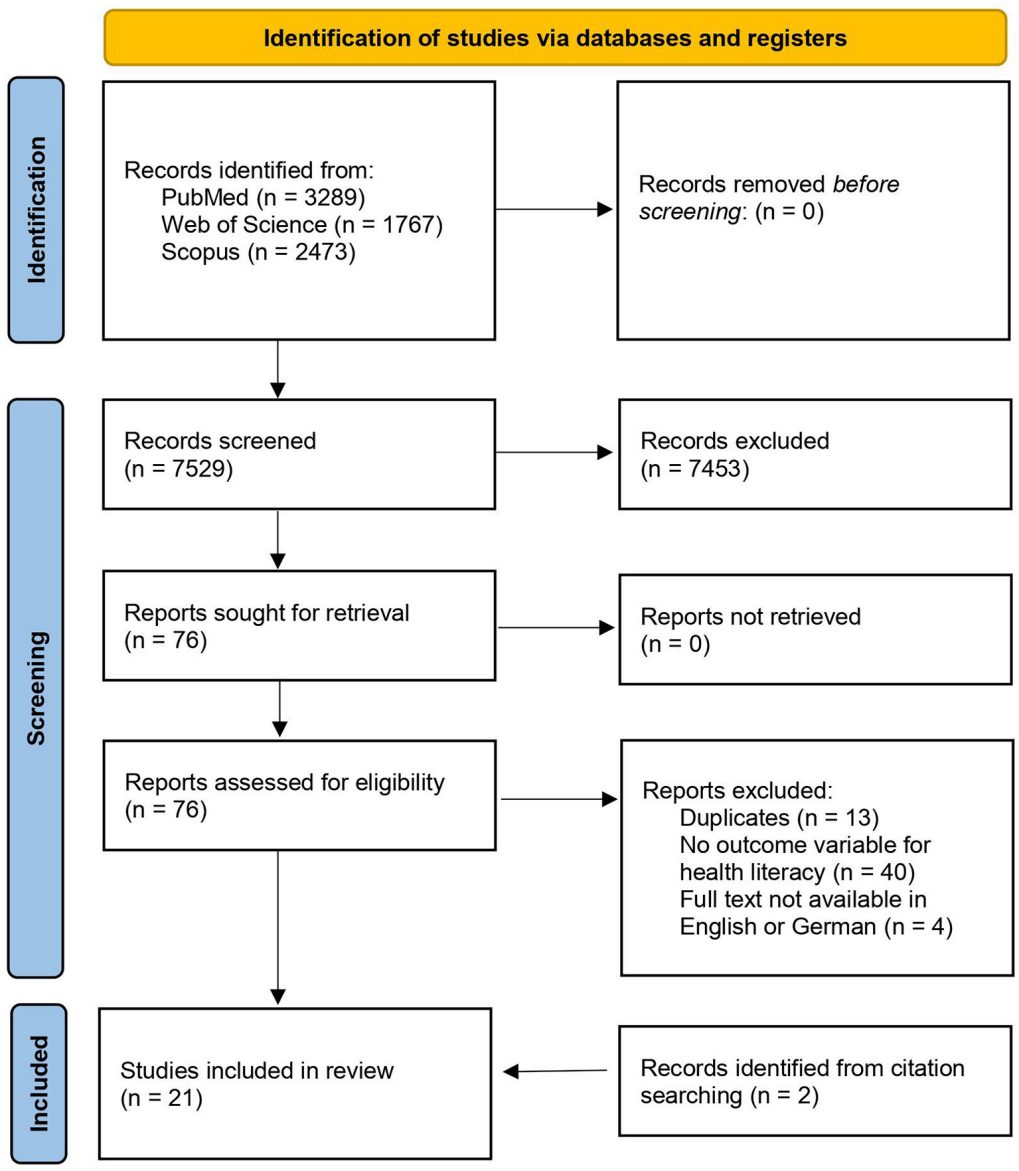
Flow diagram.

The search in the databases PubMed, Scopus, and Web of Science resulted in a total of 7,529 hits with the selected search terms. Out of those, 7,139 studies were excluded due to an inappropriate title, indicating an obviously different topic. Another 314 studies were excluded after the abstract review because they did not meet the necessary inclusion criteria. Thirteen further studies were removed after testing for duplicates. The full texts of the remaining 63 studies were then reviewed in detail. Forty-four of these did not meet the specified inclusion criteria. The remaining 19 studies were deemed suitable for inclusion in the review. In addition, further two studies could be identified by searching the references of these studies. Thus, a total of 21 studies were finally included in the review. Figure 1 presents a flow diagram summarizing the selection process.

Seventeen studies were published in English and four in German. Studies had been conducted in Taiwan, Jordan, Denmark, the United States of America, Laos, Germany, Iran, Nepal, Portugal, Australia, Singapore, Lithuania, China, and Turkey. The selected studies were published in the period from 2015 to 2019. The included studies involved 13,772 students in higher education settings with the smallest sample size of 37 students and the biggest sample size of 2,892 students. The mean age of the students ranged from 20.1 to 24.1 years for the studies where data were available. Regarding student groups, twelve studies included students from various study programs, seven studies included students from various health-related study programs, and two studies included only one specific health-related program. Of the included studies, 17 were conducted in universities and two in colleges. Two studies provided no information about the setting. Theoretical frameworks for health literacy were the definition of the WHO (10), Nutbeam (21), Sørensen et al. (12), Baker (27), Kickbusch and Maag (28), Kickbusch, Maag, and Wait (29), Paasche-Orlow and Wolf (30), and Zarcadoolas, Pleasant, and Greer (31). Various scales were used to assess health literacy: The Turkey Health Literacy Scale (32), the Perception of Health Scale (33), the Health Literacy Questionnaire (34), the Danish version of the Health Literacy Questionnaire (35), concepts of Wieland and Hammes (36), Bässler (37), and Woll (38), the Iranian Health literacy questionnaire (39), the questionnaire of health-promoting lifestyle profile II (40), short version of the Test of Functional Health Literacy in Adults (S-TOFHLA) (41), the European Health Literacy Survey Questionnaire (HLS-EU)-Q16 (42), the HLS-Asia questionnaire (43), the HLS-EU-Portugal (PT) (44), The eHealth Literacy Scale (eHEALS) (45), the Taiwanese eHealth literacy scale (46), the dietary behaviors scale (47), and several self-made scales. With the exception of the performance-based S-TOFHLA (41), and a performance-based interview used by Kushalnagar et al. (48), these are all so-called self-reported health literacy instruments, i.e., instruments in which subjects are asked to self-assess their abilities (49). The survey instruments are largely based on rather broader definitions of health literacy and thus go beyond the functional aspect of it. The WHO definition is used as the theoretical basis in the Health Literacy Questionnaire (HLQ). The definition and model of Norman and Skinner (19) form the basis for eHEALS (45). Several different survey instruments are supported by the theoretical model of Sørensen et al. (12). The study by Kushalnagar et al. (48) also used its own survey instruments on the theoretical basis of Baker (27) and Nutbeam (21). Göring and Rudolph (50) assessed health literacy using a survey instrument based on the theory of Wieland and Hammes (36). The conceptual framework of the survey instrument used by Kaboudi et al. (51) was based on the theoretical considerations of Ratzan et al. (52).

In the study of Birimoglu and Cagalar (53), the health literacy of nursing students was insufficient compared to the data of other studies. Furthermore, working parents were associated with higher health literacy levels. Most students in the study by Budhathoki et al. (54) had only moderate health literacy and few individuals reported high health literacy according to their mean scores on the HLQ (34) scales. Thereby, higher age, being enrolled in a health-related course of study, higher educational level of parents, and male sex were associated with higher levels of health literacy. Elsborg et al. (55) showed that the health literacy scores of students were higher than the scores of the Danish population. Here, a higher study semester, female sex, being enrolled in a health-related course of studies, a higher educational level of the parents, and health-related experiences had a positive correlation with health literacy. The results of Göring and Rudolph (50) indicate that higher sports activity and male sex correlate positively with higher health literacy. Moreover, a finding of the study is that the mean health literacy values of common students are below the values of vocational school students. Kaboudi et al. (51) stated that in their study the mean and SD of the total health literacy of students were 4.04 ± 0.43 out of a score of five on the Iranian Health Literacy Questionnaire (39), indicating good health literacy. They found that healthy behavior is positively correlated with high health literacy. Due to their specific sample and measurement tools, Kushalnagar et al. (48) made no statement regarding the overall health literacy scores of deaf college students. The data showed a strong relationship between greater frequency of health-related discussions with friends and an accessible language during childhood and higher critical health literacy scores.

The results of Mullan et al. (56) suggest that different student groups have different health literacy profiles due to medical students demonstrating higher health literacy than students from other health-related courses of studies. Nevertheless, the authors conclude that students who are enrolled in a health-related course of studies, particularly nursing students, have gaps regarding their health literacy based on low to medium mean scores for the different HLQ (34) scales. Rababah et al. (57) also found limitations of health literacy among college students comparing the collected mean scores of the HLQ (34) to levels reported in the study of the measurement tool. Apart from the negative impact of smoking, health literacy was positively associated with higher age, higher study semester, female sex, and enrollment in a health-related course of studies. Compared with other population groups in Germany, there are more students with problematic health literacy according to Reick and Hering (58). Ninety-three percent of students in a study by Runk et al. (59) were found to have less than sufficient health literacy based on a reference index. According to the authors, accessible health services in the population and social understanding of health and disease and media distribution positively correlate with high health literacy levels. Santos et al. (60) made no statement regarding overall health literacy due to their specific research question, but found the internet as a poor source for information gathering among students. Compared to the adult population of North-Rhine-Westphalia and the German general population, students surveyed by Schricker et al. (61) have shown lower health literacy levels. While a higher subjective social status was positively correlated with the score, unfavorable financial situation and limited social support were negatively associated with health literacy by the authors. More than half of the students in the study by Schultes (62) have a high level of health literacy but are below the average in a European country comparison. Health-promoting behaviors of subjective health assessment and daily fruit and vegetable consumption were associated with better health literacy levels. The health literacy levels of the students in the study by Sukys et al. (63) were either lower, similar, or higher depending on international reference studies. A positive correlation with health literacy was found with the female sex and with enrollment in health-related courses. Suri et al. (64) did not make a statement regarding general health literacy in their study. Their work focused on the influence of the type of information gathering (traditional sources vs. internet) on health literacy and underlines that different domain-specific health literacy skills for different health sources are needed. According to Vamos et al. (65), there is a gap in health literacy among the sample groups based on the mean scores for the different HLQ (34) domains. In their data, older age, female sex, higher parental education, and higher socioeconomic status are associated with higher health literacy levels.

The general student population in the study by Zhang et al. (66) achieved a mean score of 131.89 ± 18.84 to the overall score of 197.00 in the HLQ (34). In addition, the data indicate that the health literacy levels of the medical students are insufficient. According to the authors, higher study semester, course of studies (engineering), higher educational level of the parents, and higher socioeconomic status are positively correlated with health literacy, while depression and anxiety disorders are negatively correlated. Zou et al. (67) described in their study that the health literacy level of the student group examined is suboptimal compared to other studies. Thereby, a higher study semester, a higher educational level of the parents, and a higher socio-economic status were associated with better health literacy levels. Yang et al. (68) made no statement regarding overall eHealth literacy but found that a medical course of study resulted in higher levels. Regarding critical eHealth literacy, a positive, health-promoting behavior was positively correlated. In the study by Luo et al. (69), eHealth literacy levels of students were medium to high due to the collected mean scores of 3.66 ± 0.70 for functional eHealth literacy, 3.67 ± 0.67 for interactive eHealth literacy, and 3.65 ± 0.69 for critical eHealth literacy each with a maximum score of five with eHEALS (45). Positive correlation for functional eHealth literacy was found with high frequency in the use of medical services, for interactive eHealth literacy with the selection of suitable types and locations and low intervals of health services utilization and for critical eHealth literacy with the selection of suitable types, locations, and purpose aspects of health services utilization. Medium-to-high levels of eHealth literacy for the student sample were described in the study by Yang et al. (70) indicated through the mean scores of functional eHealth literacy with 3.56 ± 0.77, interactive eHealth literacy with 3.57 ± 0.71, and critical eHealth literacy with 3.59 ± 0.72 out of a maximum score of five with the eHEALS measurement tool (45). Additionally, functional eHealth literacy was negatively related to unhealthy food intake, interactive eHealth literacy was positively related to a balanced diet, and critical eHealth literacy was positively related to regular eating habits. Also, interactive eHealth literacy and critical eHealth literacy were positively correlated with positive attitudes and decisions about food purchasing. Table 1 presents the results regarding the general levels of health literacy and possible determinants of these.

**Table 1:** Results of individual studies.

**Reference**	**Participants Gender Mean age**	**Facilities**	**Theoretical frame(s)**	**Scales used**	**Possible determinants^a^**
Suri et al. (64)	1,062 students of all courses ♂46.3% ♀53.7% no mean age available (range: 18–38+)	Large University, Singapore	Zarcadoolas et al. (31)	Parts of HLQ^a^, eHEALS^c^	**[+/-]** type of information gathering: traditional sources vs. internet (different domain-specific health literacy skills for different health sources)
Vamos et al. (65)	221 students from courses related to business administration, science and arts, nursing, education and human development ♂33.5% ♀66.5% 27 (median) (range: 15–30+)	University in southern Texas, USA	Kickbusch, Wait and Maag (29); Paasche-Orlow and Wolf (30); WHO (10); Sørensen et al. (12)	HLQ	**[+]** higher age **[+]** female gender **[+]** higher educational level of the parents **[+]** higher socioeconomic status
Zhang et al. (66)	1272 students of health-related courses ♂19.7% ♀80.3% 15 −19J.0 39.9%; 20.24 J. 59.9%, ab 25 J. 0.2% no mean age available (range: 15–30+)	Medical University in Chongqing, China	Sørensen et al. (12)	HLQ	**[+]** higher study semester **[+]** course of studies: engineering **[+]** higher educational level of the parents **[+]** higher socioeconomic status **[-]** depression / anxiety disorders
Elsborg et al. (55)	376 students of health-related courses ♂27.1% ♀72.9% no mean age available (range: 15–30+)	Several Universities in Denmark, Denmark	WHO (10); Sørensen et al. (12)	HLQ	**[+]** higher study semester **[+]** female Gender **[+]** course of studies: health-related **[+]** higher educational level of the parents **[+]** health-related experiences (e.g., hospital stay)
Kaboudi et al. (51)	420 students of health-related courses ♂47.6% ♀52.4% 22.50 (SD = 2.22)	Kermanshah University of Medical Sciences, Iran	Baker (27); WHO (10)	IHLQ^d^, HPLP-II^e^	**[+]** health-promoting behavior
Mullan et al. (56)	371 students of health-related courses ♂36% ♀61% 25 (median)	University of Wollongong, Australia	Sørensen et al. (12); WHO (10); Nutbeam (21)	HLQ	**[+]**course of studies: medical students
Budhathoki et al. (54)	419 students of health-related courses ♂55.8% ♀44.2% no mean age available (range: 15–25+) (68.3% ≤ 19 *years*)	University: B.P. Koirala Institute of Health Sciences (BPKIHS), Nepal	Nutbeam (21)	HLQ	**[+]** higher age **[+]** course of studies: health-related **[+]** higher educational level of the parents **[+]** male gender
Zou et al. (67)	615 undergraduate nursing students ♂9.4% ♀90.6% no mean age available (range: 15–24)	Medical University in Chongqing, China	Baker (27); Nutbeam (21); Sørensen et al. (12)	HLQ	**[+]** higher study semester **[+]** higher educational level of the parents **[+]** higher socioeconomic status
Rababah et al. (57)	520 students of health-related and other courses ♂47.5% ♀52.5% 21.03 (SD = 2.29)	Jordan University of Science and Technology, Jordan	WHO (10); Sørensen et al. (12)	HLQ	**[+]** higher age **[+]** higher study semester **[+]** female gender **[+]** course of studies: health-related **[-]** smoking
Schultes (62)	533 bachelor students from four different courses of studies ♂29% ♀71% no mean age available (range: <19–29)	University of Applied Sciences, Hochschule Fulda, Germany	Kickbusch et al. (29)	HLS-EU-Q16^f^	**[+]** health-promoting behavior: Subjective health assessment **[+]** health-promoting behavior: Daily fruit and vegetable consumption
Runk et al. (59)	244 students from courses: environmental sciences and business administration and economics ♂39.3% ♀60.7% 19.7 (range: 17–29)	National University of Laos PDR, Laos	Nutbeam (21); Sørensen et al. (12);Zarcadoolas et al. (31); Zarcadoolas et al. (2003, 2005)	HLS-Asia^g^; interviews	**[+]** accessible health services in the population and social understanding of health and disease **[+]** media distribution
Sukys et al. (63)	912 students of all courses ♂63.3% ♀36.7% 21.08 (SD = 1.42)	Universities in Kaunas, Klaipeda and Vilnius, Lithuania	Sørensen et al. (12)	HLS-EU-Q47^h^	**[+]** female gender **[+]** enrollment in health-related courses
Reick and Hering (58)	127 students of health-related courses ♂7.9% ♀89.7% 24.1 (SD = 5.5)	University of Applied Science: Hochschule für Gesundheit Bochum, Germany	Sørensen et al. (12)	HLS-EU-Q16	None
Santos et al. (60)	485 students of all courses ♂22.5% ♀77.5% 23 (median)	University of Porto, Portugal	Nutbeam (21); Sørensen et al. (12)	HLS-EU-PT^i^	**[-]** using internet for information gathering
Birimoglu and Cagalar (53)	409 nursing students ♂37.7% ♀62.3% 20.81 (SD = 2.1)	University in Hatay, Turkey	WHO (10); Sørensen et al. (12)	THLS-32^j^; PHS^k^	**[+]** working parents
Schricker et al. (61)	996 students of all courses ♂30.1% ♀69.8% 22.80 (SD = 3.09)	TU Dortmund University, Germany	Sørensen et al. (12)	HLS-EU-Q16	**[+]** higher subjective social status **[-]** unfavorable financial situation **[-]** limited social support
Yang et al. (68)	556 college students of all courses ♂19.1% ♀80.9% age: no data	14 Colleges in Taiwan	Nutbeam (21)	eHEALS; HPLS^l^	**[+]** course of studies: medical (only in terms of ehealth literacy) **[+]** positive, health-promoting behavior (only in terms of critical ehealth literacy)
Luo et al. (69)	489 college students of all courses ♂37.4% ♀62.6% 21.51 (SD = 4.11)	9 Colleges in Taiwan	Nutbeam (21)	eHEALS; HSUS^m^	**[+]** high frequency in the use of medical services (only in terms of functional ehealth literacy) **[+]** selection of suitable types and locations and low interval of health services utilization (only in terms of interactive ehealth literacy) **[+]** selection of suitable types, locations and purpose aspects of health services utilization (only in terms of critical ehealth literacy)
Yang et al. (70)	813 college students of all courses ♂52.9% ♀47.1% 20.08 (SD = 1.43)	10 Colleges in Taiwan	Nutbeam (21)	eHEALS; DBS^n^	**[+]** less intake of unhealthy food (only in terms of functional ehealth literacy) **[+]** balanced diet and health aspects of consumers' nutritional behavior (only in terms of interactive ehealth literacy) **[+]** regular eating habits and consumer health (only in terms of critical ehealth literacy)
Göring and Rudolph (50)	2892 students of all courses ♂34.5% ♀65.5% 23.4 (SD/range: no data)	Georg-August-University Göttingen, Germany	WHO (10); Nutbeam (21); Kickbusch and Maag (28)	GKF^o^; typification of sports activity Bässler (37) and Woll (38)	**[+]** higher sports activity **[+]** male gender
Kushalnagar et al. (48)	37 deaf undergraduate college students of all courses ♂45.9% ♀54.1% 22.38 (SD = 2.68)	American college(s), USA	Nutbeam (21); Sørensen et al. (12)	S-TOFHLA^p^, self-developed instruments, interviews	**[+]** greater frequency of health-related discussions with friends (only in terms of critical health literacy) **[+]** accessible language during childhood (only in terms of critical health literacy)

a *“**[+]”**: promoting determinant; **“[-]”**: inhibiting determinant*.

b *Health Literacy Questionnaire*.

c *eHealth Literacy Scale*.

d *Iranian Health Literacy Questionnaire*.

e *Questionnaire of health-promoting lifestyle profile II*.

f *Short form of the European Health Literacy Questionnaire (HLS-EU)*.

g *Health Literacy Survey Asia: Version of the HLS-EU for Asia and the Pacific*.

h *European Health Literacy Questionnaire (HLS-EU)*.

i *Portuguese version of the HLS-EU*.

j *Turkish version of the HLS-EU: Turkey Health Literacy Scale (THLS-32)*.

k *Perception of Health Scale (PHS)*.

l *Health-promoting Lifestyle Scale*.

m *Health Services Utilization Scale*.

n *Dietary Behaviors Scale*.

o *Questionnaire for Health Literacy Expectation (german): Fragebogen zur Gesundheitskompetenzerwartung (GKF), Wieland and Hammes (36)*.

p *Short Test of Functional Health Literacy in Adults*.

*♂ = male sex; ♀ = female sex*.

To compile and interpret the results of the studies in a meaningful way, it is important to consider differences and similarities, especially in terms of the methods used. As these are exclusively cross-sectional studies, all studies are relatively homogeneous regarding study design. The greatest differences can be found in the selected samples (several health-related courses of study vs. one specifically health-related course of study vs. various courses of study and number of semesters) and the measuring instrument used. The results of the examined studies show a relatively homogeneous picture regarding their data on the health literacy of students. Eleven studies (50, 53, 54, 56–59, 61, 65–67) report poor values or limited health literacy among students. A total of 8,089 students were involved in these studies. Regarding the study course, there is an even distribution between explicitly health-related and various study programs. Five studies include several health-related and five studies include all study programs. Only one study focuses on undergraduate nursing students solely.

For five studies, information on the number of semesters was available. Two studies explicitly included all semesters and three focused on students at the beginning of their study careers. These distributions about the course of study and the number of semesters must be taken into account when considering the results. The measuring instruments used in the studies are all assessed as valid and reliable, except for Göring and Rudolph (50), who used a self-made measuring instrument. The measurement instruments used were considered valid and reliable if they were sophisticated health literacy measurement instruments (e.g., HLQ) that had been previously tested, piloted, and repeatedly published.

The statements made on the health literacy of students are justified in each study due to comparisons with other populations. In fact, only two studies (51, 55) report higher health literacy scores among students than among the national population. A total of 796 students were surveyed in the two studies with reliable and valid HLQ. It should be noted that these are exclusively health-related programs and therefore their results should be interpreted accordingly. The results of one of the studies were compared with the Danish rural population and the results of the second study with older studies and with a reference sample.

In the studies of Schultes (62) and Sukys et al. (63), no conclusion regarding the results was reached since the comparison with different reference samples brought different results. The long and the short form of the HLS-EU was used for measurement in both of these studies. In the study by Schultes (62), various bachelor's degree programs were included and in the study by Sukys et al. (63) different health study programs, except for medicine. In other three studies (48, 60, 64), no conclusion regarding general health literacy is given. Regarding eHealth literacy, authors of two studies (69, 70) speak of medium or higher scores based on a score of their measurement instrument, and the third study (68) made no statement regarding general eHealth literacy levels. It should be noted that these three studies were conducted by the same research team.

Quantifying the risk of bias of the included studies using the AXIS tool (see Table 2), seven studies were classified as very low risk of bias (54, 56, 57, 64, 66, 67, 69), 11 studies were classified as low risk of bias (48, 50, 51, 55, 58, 60, 61, 63, 65, 68, 70), two studies were classified as the moderate risk of bias (53, 59), and one study was classified as high risk of bias (62). In terms of quality, we are therefore dealing with a comparatively solid and homogeneous study situation, with only three out of 21 studies falling short. The main weaknesses of the included studies were the lack of sample size justification and not addressing non-responders.

**Table 2:** Quality assessment of the included studies.

	**Kushalnagar et al. (48)**	**Göring and Rudolph (50)**	**Kaboudi et al. (51)**	**Birimoglu Okuyan and Caglar (53)**	**Budhathoki et al. (54)**	**Elsborg et al. (55)**	**Mullan et al. (56)**	**Rababah et al. (57)**	**Reick and Hering (58)**	**Runk et al. (59)**	**Santos et al. (60)**	**Schricker et al. (61)**	**Schultes (62)**	**Sukys et al. (63)**	**Suri et al. (64)**	**Vamos et al. (65)**	**Zhang et al. (66)**	**Zou et al. (67)**	**Yang et al. (68)**	**Luo et al. (69)**	**Yang et al. (70)**
Q1	1	1	1	1	1	1	1	1	1	1	1	1	1	1	1	1	1	1	1	1	1
Q2	1	1	1	1	1	1	1	1	1	1	1	1	1	1	1	1	1	1	1	1	1
Q3	0	0	1	0	1	0	1	1	0	0	1	0	1	0	1	1	1	1	0	1	0
Q4	1	1	1	1	1	1	1	1	1	1	1	1	1	1	1	1	1	1	1	1	1
Q5	1	1	1	1	1	1	1	1	1	1	1	1	1	1	1	1	1	1	1	1	1
Q6	1	1	1	1	1	1	1	1	1	1	1	1	1	1	1	1	1	1	1	1	1
Q7	0	1	0	0	1	0	1	1	1	0	0	1	0	1	1	1	1	1	1	1	1
Q8	1	1	1	1	1	1	1	1	1	1	1	1	1	1	1	1	1	1	1	1	1
Q9	1	1	1	1	1	1	1	1	1	1	1	1	1	1	1	1	1	1	1	1	1
Q10	1	1	1	1	1	1	1	1	1	1	1	1	0	1	1	1	1	1	1	1	1
Q11	1	1	1	1	1	1	1	1	1	1	1	1	0	1	1	1	1	1	1	1	1
Q12	1	1	1	1	1	1	1	1	1	1	1	1	1	1	1	1	1	1	1	1	1
Q13	1	1	1	1	1	1	1	1	0	1	1	1	1	1	1	0	1	1	1	1	1
Q14	0	1	0	0	0	0	0	0	1	0	0	0	0	0	0	0	0	0	0	0	0
Q15	1	1	1	1	1	1	1	1	1	1	1	1	1	1	1	1	1	1	1	1	1
Q16	1	1	1	1	1	1	1	1	1	1	1	1	0	1	1	1	1	1	1	1	1
Q17	1	1	1	1	1	1	1	1	1	1	1	1	1	1	1	1	1	1	1	1	1
Q18	1	1	1	0	1	1	1	1	1	1	1	1	0	1	1	1	1	1	1	1	1
Q19	1	1	1	1	1	1	1	1	1	1	1	1	1	1	1	1	1	1	1	1	1
Q20	1	0	1	1	1	1	1	1	0	0	1	1	1	1	1	1	1	1	1	1	1
**Score**	**17**	**18**	**18**	**16**	**19**	**17**	**19**	**19**	**17**	**16**	**18**	**18**	**14**	**18**	**19**	**18**	**19**	**19**	**18**	**19**	**18**

*1, criterion met; 0, criterion not met*.

### Possible Determinants of Health Literacy

Among the determinants presented, there was strong evidence for a relationship between health literacy and age, the semester of study, gender, course of studies, parental education, and socioeconomic background. Other possible determinants could be accessed to information, health-related experiences, financial situation, social support, housing situation, physical activity, smoking status, symptoms of depression and anxiety disorders, employment status of parents, and daily fruit and vegetable consumption. For students with impaired hearing or deafness, the frequency of health-related discussions with friends and access to a language in childhood play a critical role. Electronic health literacy may be related to a medical degree course. There are also several determinants for the respective sublevels of eHealth literacy. With regard to the length of this section, the methodology and conduct of individual studies are only discussed, if they involve a special sample or use a debatable measuring instrument.

### Age

Better health literacy with increasing age is shown in three studies (54, 57, 65) with 1,160 students overall, of which 419 come from health professional training programs (54). This correlation can be explained by increased experience with the healthcare system. With increasing age and experience, older students have an advanced ability to navigate within the healthcare system and engage with healthcare professionals. This results in increased awareness of health promotion resources in their environment and greater self-confidence when talking to healthcare professionals (54, 65). One study with 127 students found no correlation between health literacy and age (58).

### Gender

In terms of gender, there were four studies (55, 57, 63, 65) with a total of 2,029 participants that measured higher health literacy among female students and two studies (50, 54) with a total of 3,311 participants that measured higher health literacy among male students, whereby it should be mentioned that Göring and Rudolph (50) used a self-made measuring instrument. Except for two studies (54, 55), these results refer to various study programs. These differences can be explained by variations in the educational system on the one hand, and sociocultural influences on the other (55, 57). For example, in predominantly patriarchal societies, women have less influence on household decision-making. Also, male children are preferred to female children because of the idea that boys need more knowledge and therefore should be able to maintain their health (54). Another explanation could also be that women assess the individual ability to influence subjective health in a different way than men. For example, a different perception of complaints and specifically female complaints can influence one's own self-efficacy expectations regarding one's health in a different way to men (50). Two studies with 1,123 participants, however (58, 61), could not find any differences between genders.

### The Course of Studies

Six studies with a total of 3,873 students overall describe different levels of health literacy concerning the course of studies (54–57, 63, 66). Except for Rababah et al. (57), these results were found in studies that compared health-related courses of study. The results must, therefore, be interpreted carefully. These results can be explained by the specificity in certain health-related curricula. The contents of multiple health-related courses of study usually cover different areas of health promotion and disease prevention and individual political and organizational health areas. Students in health settings overall have better access to and understanding of health-related information, which facilitates decision-making and application of the decision. Besides, students in health-related courses of study often have a personal interest in the context of health promotion and the associated competencies due to their choice of study (54, 55, 63).

### Study Semester

As the number of semesters of health students increases, so do the values of health literacy according to four studies (55, 57, 66, 67) with a total of 2,783 participants. This supports the assumption that in addition to personal motivation, the curriculum has a major influence on acquiring skills and knowledge related to one's health. As the semester increases, so does the knowledge obtained. Late semesters already have more medical expertise and know-how to obtain quality information (55, 66, 67). One study with 127 students found no correlation regarding this determinant (58).

### Parental Education

Five studies including a total of 2,903 students recorded higher health literacy if their parents have received higher education (54, 55, 65–67). Except for Vamos et al. (65), this concerns students from several health-related courses. Possible explanations could be the increased health awareness of the parents due to their education, which enables them to navigate their children through the health system and rubs off on the children (54, 55, 65–67). One study with 127 participants found no correlation between the education of parents and the health literacy of students (58).

### Socioeconomic Background

Three studies including a total of 2,108 students found that higher socioeconomic groups have better access, understanding, and handling of health-related resources (65–67). Within this result, all three forms of existing samples are present (several health-related courses of study, one specifically health-related course of study, various courses of study, and the number of semesters). Due to their higher socioeconomic status, students are more likely to be exposed to or have access to relevant information from parents and other health promotion resources. Here too, parents play a decisive role, since the socioeconomic status of students reflects the socioeconomic status of their parents (65, 67).

### Access to Information

One study (60) with 485 students from all courses of the study found that while the internet is the most popular way for students to access information, it is also associated with the worst health literacy scores (compared to those, who appeal to family and friends or specialty journals as a source of health information). This is most likely due to the quality of information available on the internet. Information on the internet is often incorrect and hardly comprehendible.

### Health-Related Experiences

According to one study (55) with a sample size of 376 participants, students in health-related programs who have already gained experience in healthcare (e.g., hospitalization) have better health literacy. The reason for this is the experience they have already had and the support they receive from healthcare providers and their assessment of their ability to find health-related information and communicate with healthcare professionals.

### Physical Activity

Regarding physical activity, one study including 2,892 students (50) from various courses of study reports a positive relationship between health literacy and sporting activity due to increased self-efficacy expectations, measured with a self-made measuring instrument. One study with 533 students (62) also from various courses of study, on the other hand, does not report any correlation, this being the study with a high risk of bias.

Various other determinants of health literacy for several health-related and various courses of study were discussed in the involved studies: better financial situation (61) and positively perceived health behavior (62), non-smoking status (57), symptoms of depression and anxiety disorders (66), and daily consumption of fruits and vegetables (62). Social support should also be mentioned, as social exchange processes can lead to greater security in obtaining and handling health-related information (61). Lastly, the employment status of parents is of interest, as higher health literacy was found among students with working parents. This phenomenon could be explained by better access to technological resources (53).

### No Influence on Health Literacy

In addition to the abovementioned missing correlations, no connection was found between health literacy and the migration background (61) or membership to a health profession (58). Contrary to another study (57), one study (62) found no correlation between higher levels of health literacy and smoking status and alcohol consumption. However, it should be noted that this is a study with a moderate risk of bias.

### Special Student Groups

One study (48) measured health literacy in a group of 37 deaf students with the S-TOFHLA for functional literacy, two extra questions for interactive health literacy, and critical health literacy via the response to a self-made video. It was found that a higher frequency of health-related discussions significantly contributes to better critical health literacy. Language barriers can be avoided by healthy-literate peers who share a common language. The critical health literacy of deaf students was not influenced by the hearing ability of family members, so other social characteristics, such as the effort of the parents to communicate with the deaf individual, encourage participation in family discussions about health (48).

### Possible Determinants of eHealth Literacy

Three studies (68–70) with a total of 1,858 students have specifically addressed determinants of eHealth. In each case, the different forms of health literacy, functional, interactive, and critical, were analyzed. According to Yang et al. (68), the only general determinant for higher eHealth literacy, in general, is belonging to a medical degree program.

### Functional eHealth Literacy

In functional eHealth, a high frequency in the use of medical services was discovered. Poor understanding of medical care directions and poor problem-solving skills may lead to ineffective care and a lack of behavioral change when new information is available (69). However, a lower intake of unhealthy food could also be associated with higher functional eHealth literacy. Students are thus able to understand the risks associated with unhealthy food and can avoid its intake in everyday life (70).

### Interactive eHealth Literacy

The selection of appropriate types and locations for health services and a low frequency of use of these have been measured at high interactive levels of eHealth literacy. Interactive eHealth literacy could help students to act independently, increase their motivation and self-confidence, thereby selecting appropriate types and locations for their health needs (69). It is also linked to a balanced diet and health aspects of consumers' dietary behaviors, as interactive eHealth literacy can lead to students actively participating in everyday activities and promoting healthy consumption patterns (70).

### Critical eHealth Literacy

The highest level of eHealth literacy is linked to three possible determinants. First, the selection of appropriate types, locations, and purpose aspects of health services, as critical eHealth literacy allows individuals greater control over life events and situations, thus enabling them to evaluate health issues, as well as risks and benefits and advocate for themselves (69). Next comes regular eating habits and consumer health. By critically evaluating electronic health information, students can filter out the advantages and disadvantages of this information and apply them to their eating habits and activities (70). Finally, positive, health-promoting behaviors are associated with higher critical eHealth literacy. Through the highest level of eHealth literacy, students can engage in health-enhancing actions through critical examination and advocating for themselves, to engage in health-enhancing actions (68).

### No Influence on eHealth Literacy

No link to eHealth literacy was found in gender and frequency of consumption of organic food. As this is an educated and age-limited group, possible gender differences may have been compensated (69). The frequency of organic food consumption is probably influenced more by perceptions of food safety than by knowledge about the food itself. Various food incidents worldwide may be the primary decision maker regardless of the level of eHealth literacy (68).

## Discussion

The general level of health literacy among university students seems to be insufficient and needs to be improved. Regarding the distribution of study courses, this observation seems to apply to both health-related and other study courses–although students from health-related study programs tend to have better health literacy. The health literacy of students is influenced by different variables. In this review, strong evidence for a relationship between health literacy and age, gender, number of semesters, course of studies, parental education, and socioeconomic background was found. These assumptions must be considered with regard to the respective samples selected. For example, regarding age and gender, more studies were represented with a general sample of students, while in course of study and parental education, more studies were represented with a sample of students studying health-related subjects. Concerning the number of semesters, only students from the health sector were represented, while concerning the socioeconomic background the distribution of students was equal among all sample types.

Students can benefit from increased health literacy for their own health. In addition to the personal added value, a social benefit can arise from health-competent multipliers in responsible positions. Besides, the results should always be considered in the context of the country's existing health system and social conception of health. Particularly concerning the results of gender differences, the cultural context must be considered. Health literacy can therefore possibly only be compared between populations if social, economic, and health systems are congruent (59). In general, however, it is recommended that universities pay more attention to the promotion of health literacy when planning the curriculum or additional offers for students. Electronic health literacy levels among students were high in the studies presented. However, this result should be interpreted with caution, as all three studies involved were conducted in the same country and possibly the same colleges and contradict the results regarding normal health literacy. A review (71) with six peer-reviewed articles and one doctoral dissertation with numbers of participants ranging from 34 to 5,030 on eHealth literacy also speaks of a high level of connection to the internet among students, but also of limited eHealth literacy. As the internet is the preferred way to obtain health information even if it does not lead to better health literacy or eHealth literacy, work is needed to promote the quality of the information and the ability of students to evaluate it (60). While the results of this review must be considered carefully, they can be used as a starting point for planning interventions and monitoring health literacy among students over the long term.

Concerning the studies, limitations in the performance of the measurements and the tests used were discovered. During the data collection process, practicability was prioritized, which meant that precision and quality had to suffer. This includes the use of incomplete questionnaires (70), or the inability to secure an appropriate, private space to take measurements (57). There were also limitations in the distribution of questionnaires. The use of social media can lead to self-selection bias and a lack of control over appropriate data (55). The self-reporting method may influence the accuracy of the results and the use of e-mail and online surveys may exclude students with low affinity to the internet (51). Some of the tests used had little or no evidence of their reliability or validity. A comparison between and within the studies is also difficult, because on the one hand HLQ-scores, for example, may not be comparable due to some scales being harder to score on (56), on the other hand, some studies used the long and other studies the more roughly measuring short form of their used test (e.g., HLS-EU-Q16 and HLS-EU-Q47). When using vignettes, participants may indicate what they think they have to indicate rather than giving their honest opinion (59). Another limitation was the exclusion of international students due to a language barrier.

The results of the study cannot readily be generalized, and its interpretation should only be applied to the respective groups of students. The reasons for this are the differences between the selected samples and the selected variables studied. For example, among the included studies there was often an uneven distribution in terms of gender or number of semesters. It should also be highlighted that some studies have examined students from various study programs and others only medical or health students. Due to a lack of time and money, very little information about the students was collected mostly. There may be other mediating or confounding variables that affect health literacy.

Also, this review is not without limitations. Overall, the quality of the included studies is sound. Nevertheless, there are three studies with moderate-to-poor study quality among them, and the majority of the high-quality studies lack sample size justification and addressing of non-responders as well. Differences regarding assessment methods, study population, and sample size hamper the comparison between the studies. Finally, it should be mentioned that only German and English language studies, and studies that have already been published or were available, were considered in this review.

### Implications for Practice

Health literacy activities should target all students. Universities should make use of their resources and offer health literacy courses for students in which content is used from disciplines available at the university (e.g., medicine, health, or psychology). Multisectoral and multidisciplinary efforts are essential in promoting health for students, since not only synergies with regard to knowledge and resources are enabled, but also access to certain student subpopulations are made possible (72). To increase effectiveness, health literacy courses should be adapted according to the different needs and characteristics of the student subgroups and should be linked to evaluative research. The internet as well as gamification approaches, in particular, can help to make interventions interesting for the selected target group. Besides, social networks can provide an easy way to reach and connect students to promote their health and eHealth literacy, why peer-to-peer programs could play a role in this context. To consider special groups of students (e.g., deaf students), care should always be taken to include a suitable form of language or exchange with health literate, accessible peers in the interventions (48). Additionally, consideration should be given to the planning process when cross-curricular activities are offered for students with different backgrounds and courses of study. When planning interventions according to specific areas of health literacy, different needs of student groups can be taken into account. Furthermore, a central website of the university could be used to communicate accurate and actionable health-related information in a way that is appropriate for the target group, as has already been done during the corona pandemic through the development of corona landing pages for students with frequently asked questions.

### Implications for Research

The results of this review suggest that students are a relevant target group for future health literacy studies. Furthermore, there is a need for appropriate measurement methods in the university setting that reflects the circumstances of the living situation for students. Additional variables (e.g., structural aspects, such as support services provided by the university) that may be possible determinants of student health literacy should be collected. Once interventions have been designed, they can be examined to determine which methods and media (despite the challenge of the fast-changing digital environment) are most effective and which determinants in the cultural and social context require particular attention. To ensure that interventions are accessible to all students on campus, more research is needed on accessibility and effectiveness for specific student groups. Appropriate tools must also be developed to regularly check the quality of information available online to counteract misinformation.

## Data Availability Statement

The original contributions presented in the study are included in the article/supplementary material, further inquiries can be directed to the corresponding author.

## Author Contributions

All authors listed have made a substantial, direct, and intellectual contribution to the work and approved it for publication.

## Funding

The study received funding by the Techniker Krankenkasse (German health insurance). This article has been funded through the Open Access Publishing Fund of the Karlsruhe Institute of Technology.

## Conflict of Interest

The authors declare that the research was conducted in the absence of any commercial or financial relationships that could be construed as a potential conflict of interest.

## Publisher's Note

All claims expressed in this article are solely those of the authors and do not necessarily represent those of their affiliated organizations, or those of the publisher, the editors and the reviewers. Any product that may be evaluated in this article, or claim that may be made by its manufacturer, is not guaranteed or endorsed by the publisher.

## Appendix

### Search Term

(college OR “college students” OR university OR universities OR student OR students OR “young adult” OR “young adults” OR adolescent OR adolescents) AND (“critical health literacy” OR “health literacy” OR “eHealth literacy” OR “functional health literacy” OR “health-related literacy” OR “health literacy education” OR “literacy programs”).
